# Incidence and impact of intracranial complications in patients undergoing extracorporeal membrane oxygenation treatment

**DOI:** 10.1016/j.bas.2026.106123

**Published:** 2026-06-11

**Authors:** Jan Wiefhoff, Friedrich Thomas Irmer, Felix Maximilian Gerach, Jan Rodemerk, Ramazan Jabbarli, Marvin Darkwah Oppong, Cornelius Deuschl, Börge Schmidt, Frank Herbstreit, Thorsten Brenner, Ulrich Sure, Laurèl Rauschenbach

**Affiliations:** a Department of Anesthesiology and Intensive Care Medicine, University Hospital Essen, University Duisburg-Essen, Essen, Germany; b Department of Neurosurgery and Spine Surgery, University Hospital Essen, University of Duisburg-Essen, Essen, Germany; c DKFZ Division Translational Neurooncology at the WTZ and German Cancer Consortium (DKTK), Partner site Essen/Düsseldorf, a partnership between DKFZ and University Hospital Essen, Essen, Germany; d Center for Translational Neuroscience and Behavioral Science (C-TNBS), University of Duisburg-Essen, Essen, Germany; e Institute for Diagnostic and Interventional Radiology and Neuroradiology, University Hospital Essen, University Duisburg-Essen, Essen, Germany; f Institute for Medical Informatics, Biometry and Epidemiology, University Hospital Essen, Essen, Germany

**Keywords:** Brain edema, Extracorporeal membrane oxygenation, Hospital mortality, Intracranial hemorrhages, Ischemic stroke

## Abstract

**Introduction:**

Extracorporeal membrane oxygenation (ECMO) is a life-saving intervention for respiratory and/or cardiac failure but is associated with high morbidity and mortality, particularly due to intracranial complications.

**Research question:**

What is the incidence, type, and impact of intracranial complications in patients undergoing ECMO treatment?

**Material and methods:**

This retrospective single-center study included ECMO patients treated at a tertiary care center between 2013 and 2023. Patients with documented intracranial hemorrhage, cerebral ischemic stroke, or diffuse hypoxic cerebral edema were included. Demographic, clinical, radiological, and outcome data were analyzed.

**Results:**

Among 775 ECMO patients, 141 (18%) developed intracranial complications, including intracerebral hemorrhage (36%), subarachnoid hemorrhage (22%), diffuse hypoxic cerebral edema (25%), ischemic stroke (14%), and subdural hematoma (4%). Secondary deterioration (p = 0.009), midline shift (p = 0.005), and pupillary abnormalities (p < 0.001), were associated with in-hospital mortality, though only pupillary abnormalities remained significant in multivariate analysis (p = 0.006). Patients with diffuse hypoxic cerebral edema were younger (p < 0.001), had lower multimorbidity (p = 0.019, p < 0.001) and disease burden (p = 0.02, p = 0.011), and showed less secondary deterioration (p < 0.001) than those with bleeding or ischemic stroke. In-hospital mortality was higher in patients with intracranial complications (77%) compared to those without (59%, p < 0.001). Most survivors revealed severe disability, with a median modified Rankin Scale score of 5 at discharge.

**Discussion and conclusion:**

Acute intracranial complications in patients undergoing ECMO are frequent, heterogeneous in presentation, and associated with poor outcomes. Pupillary abnormalities emerged as the strongest predictor of mortality. Early identification of high-risk patients may facilitate targeted monitoring and timely interventions to improve outcomes.

## Highlights

• 18% of ECMO patients developed intracranial complications.
• In-hospital mortality among affected patients was 77%.
• Most survivors had severe disability at discharge (median mRS 5).
• Pupillary abnormalities were the strongest mortality predictor.
• Early neuromonitoring of high-risk patients might improve outcome.

## 1 Introduction

Extracorporeal membrane oxygenation (ECMO) is a well-established intensive care technique used to treat severe respiratory and/or cardiac failure in specialized centers (Broman et al., 2019). Its benefits have been demonstrated in multiple studies, most notably during the severe acute respiratory syndrome coronavirus 2 (SARS-CoV-2) pandemic (Zhai et al., 2023; Bertini et al., 2024; Jiang et al., 2025). However, due to the severity of the underlying conditions and the invasive nature of ECMO therapy, patients are at high risk of complications, including infections, hypoxia, thromboembolic events, and bleeding (Herrmann et al., 2022).

Intracranial complications, such as intracranial hemorrhages, cerebral ischemic strokes, and diffuse hypoxic cerebral edema, are particularly concerning in ECMO patients (Jin et al., 2023; Migdady et al., 2020). These conditions are often detected late due to sedation and are associated with high mortality and severe disability. The incidence of intracranial hemorrhages in ECMO patients ranges from 1.8% to 21%, with mortality rates between 32% and 100% (Fletcher-Sandersjöö et al., 2018). Cerebral ischemic strokes occur in 1.7% to 15% of cases, with mortality rates ranging from 60% to 95% (Iacobelli et al., 2021; Xie et al., 2017; Fletcher-Sandersjöö et al., 2017; Omar et al., 2016; Lorusso et al., 2016, 2017; Le Guennec et al., 2018). Patients who develop these complications require significantly longer intensive care unit (ICU) stays, have a higher likelihood of being discharged to long-term care facilities, and generate substantially higher healthcare costs (Nasr and Rabinstein, 2015).

Research on intracranial complications in ECMO patients is challenging due to the high variability in patient characteristics, underlying conditions, and dynamic changes during prolonged ICU treatment. Moreover, differences in clinical management across centers further complicate data interpretation. As a result, studies on this topic are scarce, often limited by small sample sizes, and characterized by a highly heterogeneous patient population. This highlights an urgent need for additional research with larger datasets to improve our understanding of these complications.

With this retrospective monocentric study, we aim to provide new insights into the incidence and outcomes of veno-venous and veno-arterial ECMO patients with intracranial complications in a high-volume, specialized tertiary care center. Additionally, we seek to identify predictors of in-hospital mortality and differences between the various intracranial complications to enhance risk assessment and optimize patient management.

## 2 Material and methods

### 2.1 Study design

Between 2013 and 2023, all patients who underwent ECMO therapy in the anesthesiology intensive care unit of our tertiary care center were retrospectively analyzed. ECMO cases were identified by screening electronic patient records for the OPS code 8-852. The cohort was then divided into two groups: patients without intracranial complications and those with intracranial complications (including hemorrhage, ischemia, and edema). This classification was based on the presence of any of the following ICD codes: I60 (subarachnoid hemorrhage), I61 (intracerebral hemorrhage), or I62 (other non-traumatic intracranial hemorrhage) for intracranial hemorrhage; I63 (cerebral infarction) or I64 (stroke not specified as hemorrhagic or ischemic) for cerebral infarction; and G93.6 (cerebral edema) for diffuse hypoxic cerebral edema. For inclusion in the intracranial pathology group, relevant clinical data had to be available in the hospital's internal documentation system, and the diagnosis had to be made during ECMO therapy. All diagnoses related to intracranial pathology were validated by an experienced neurosurgeon and neuroradiologist prior to subsequent data analysis. Patients who underwent pre-ECMO therapy without subsequent cannulation (OPS code 8-852.1) or those who transitioned between veno-venous and veno-arterial ECMO during treatment were excluded. In the rare cases where a patient received multiple ECMO cycles, only the cycle associated with the occurrence of an intracranial pathology was included in the analysis. This manuscript was prepared in accordance with the STROBE guidelines for observational studies.

### 2.2 Data acquisition

Patient data were collected in a pseudonymized form and subsequently anonymized for statistical analysis. Since all data were anonymized, informed consent was not required. Any information that could allow patient identification was excluded. The study was conducted in accordance with the principles of the Declaration of Helsinki and approved by the local ethics committee (reference number: 24-120884-BO, 10.10.2024). The study was also registered in a national clinical trial database (DRKS-ID: DRKS00035277).

Data were extracted from the hospital's internal documentation system. For the cohort without intracranial complications, only in-hospital mortality was recorded. For the cohort with intracranial complications, the following parameters were collected: sex, age, height, weight, smoking status, pre-existing conditions, Simplified Acute Physiology Score (SAPS), survival during ECMO therapy, survival in the ICU, survival at hospital discharge, duration of ECMO therapy, ICU length of stay, ECMO settings, ECMO indication, initiation time of ECMO therapy, cannulation site, neurosurgical interventions, intracranial and non-neurological complications, onset of early intracranial complications after ECMO initiation, presence of pupillary abnormalities, and neurological status at discharge. The term “myocardial infarction” encompassed isolated troponin elevation as well as ST-segment elevation myocardial infarctions (STEMI) and non-ST-segment elevation myocardial infarctions (NSTEMI). The degree of pre-existing morbidity was assessed using the Charlson Comorbidity Index (CCI). For SAPS scores, the highest recorded value (SAPS peak) was used. The neurological condition at discharge was quantified using the modified Rankin Scale (mRS). Available CT imaging was analyzed for the following parameters: intracranial localization of complications, secondary deterioration (defined as an increase in hemorrhage, infarct, or diffuse hypoxic edema volume, or as the occurrence of additional intracranial complications), and midline shift.

### 2.3 In-hospital management of ECMO patients

All ECMO patients admitted to the anesthesiology intensive care unit were treated according to a standardized protocol that included comprehensive laboratory testing, microbiological screening, and ultrasound examination upon admission. The initial diagnostic workup routinely included CT imaging of the brain, thorax, and abdomen, as well as invasive monitoring via an arterial line and a pulmonary artery catheter, transesophageal echocardiography, and microbiological sampling. If necessary, invasive ventilation was performed using Biphasic Positive Airway Pressure (BIPAP) mode (Dräger Evita, Dräger, Lübeck, Germany), with the inspiratory-to-expiratory (I:E) ratio set to ensure adequate expiration and prevent air trapping. A best Positive End-Expiratory Pressure (PEEP) trial was conducted using PEEP levels above the lower inflection point identified on the low-flow pressure/volume (P/V) loop. Tidal volume was set to 6 mL/kg (ideal body weight), and driving pressure was maintained below 15 mbar. Nitric oxide (NO) up to 40 ppm was available for the treatment of right heart failure and pulmonary hypertension. In cases of acute respiratory distress syndrome (ARDS), patients were placed in a prone position (135°, with the better lung facing downward) for 16 h per day, typically for at least three days in responders. ECMO therapy was conducted using the Cardiohelp System (Getinge Deutschland GmbH, Rastatt, Germany) with heparin-coated (Bioline Coating) tubing (Cardiohelp HLS Set Advanced 7.0; Getinge Deutschland GmbH, Rastatt, Germany) and a heater (HU35; Getinge Deutschland GmbH, Rastatt, Germany). Indications for ECMO and the medical management during ECMO therapy were previously described by Hilder et al. (2017) and Herbstreit et al. (2021).

Our institution operates an established regional ECMO retrieval program, allowing ECMO therapy to be initiated both at our tertiary care center and at referring hospitals prior to patient transfer. To ensure continuous availability, a dedicated retrieval service was maintained around the clock. Retrieval missions were performed by a specialized ECMO team consisting of at least two anesthesiologists, including one board-certified intensivist. All team members were experienced in ECMO cannulation, transport, and critical care management and fulfilled the regulatory requirements for interhospital transport of critically ill patients. During ECMO therapy, neurological examinations were performed three times daily (once per shift) by the attending intensive care physician. Follow-up cranial imaging was obtained in cases of pupillary abnormalities, seizures, or a diminished level of consciousness following sedation withdrawal. In cases where CT imaging revealed an intracranial pathology, a neurosurgical consultation was requested. Depending on the severity and location of the pathology, a decision was made regarding conservative versus surgical treatment - unless a poor prognosis was confirmed, in which case best supportive care was initiated. For conservatively treated patients, a follow-up CT scan was performed after 6 h. In surgically treated cases, an immediate postoperative follow-up CT scan was obtained.

All patients on ECMO therapy were routinely anticoagulated and received continuous unfractionated heparin; argatroban was used in cases of confirmed or suspected heparin-induced thrombocytopenia (HIT). Coagulation status was monitored by measuring activated partial thromboplastin time (aPTT) every 6 h. The aPTT (reference range: 24.4–32.4 s) was maintained between 40 and 50 s during veno-venous ECMO and between 50 and 60 s during veno-arterial ECMO. Platelet counts (normal range: 180–380/nL) were maintained above 50/nL. In the event of a diagnosed bleeding complication, anticoagulation therapy was generally discontinued, and coagulation factors were administered as needed to achieve full normalization of coagulation. Only in cases involving mild intracranial pathology and a critical need for anticoagulation was full normalization occasionally omitted. To prevent thrombosis after the cessation of anticoagulation, intermittent pneumatic compression were applied.

### 2.4 Statistical analysis

Data collection and statistical analysis were performed using SPSS software (version 29.0.2.0), with data visualization facilitated by PRISM-9. Univariate analyses were conducted to identify predictors of patient outcomes. For continuous and normally distributed variables, the Student's t-test was used. For dichotomous variables, the Chi-square test was used when sample sizes exceeded five, while the Fisher exact test was applied for smaller sample sizes (≤5). Odds ratios (OR) and 95% confidence intervals (CI) were calculated to evaluate associations between specific factors and in-hospital survival. Multivariate analyses were performed using a binary logistic regression model. For subgroup analyses, the Kruskal-Wallis test was applied.

## 3 Results

### 3.1 Patient cohort

A total of 1164 records were identified using the OPS code 8-852. Among 775 patients who underwent ECMO therapy, 141 (18%) presented with intracranial complications detectable on cranial CT imaging and were included in the final analysis. The cohort had a mean age of 48 ± 15 years, with an equal gender distribution (70/141, 50% female). The mean BMI was 31 ± 9 kg/m^2^, and a history of smoking was documented in 16% (22/141) of patients. The median CCI was 2 (IQR: 0–3). In the overall cohort, in-hospital mortality was 62% (482/775), with 59% (374/634) in patients without intracranial complications and 77% (108/141) in those with such complications. Detailed data are presented in Table 1.

**Table 1:** **Baseline patient characteristics and clinical outcomes**.

Item	Calculation
Female sex (N, %)	70/141 (50)
Age (years, mean ± SD)	48 ± 15
Height (cm, mean ± SD)	171 ± 11
Weight (kg, mean ± SD)	91 ± 30
BMI (kg/m^2^, mean ± SD)	31 ± 9
Active smoker (N, %)	22/141(16)
CCI (median, IQR)	2 (0-3)
SAPS_peak_ (median, IQR)	52 (44-60)
Survival ECMO therapy (N, %)	50/141 (36)
Survival ICU (N, %)	35/141 (25)
Survival hospital (N, %)	33/141 (23)
Length of ECMO therapy (days, mean ± SD)	13 ± 15
Length of ICU stay (days, mean ± SD)^1^	21 ± 27

Note: 1, after the start of ECMO therapy; N, number; SD, standard deviation; BMI, Body-Mass-Index; CCI, Charlson Comorbidity Index; SAPS_peak_, Peak of Simplified Acute Physiology Score; ECMO, extracorporeal membrane oxygenation; ICU, intensive care unit.

### 3.2 ECMO-related patient data

Most patients (119/141, 84%) received veno-venous ECMO, while 22 (16%) underwent veno-arterial ECMO. The most common ECMO indication was ARDS due to pneumonia (95/141, 67%), followed by extracorporeal life support (16/141, 11%). Other indications included aspiration, COPD exacerbation, trauma, cardiogenic shock. Among pneumonia cases, viral etiologies were identified in 62 of 95 patients, with COVID-19 (46/95, 48%) and Influenza A (14/95, 15%) as the most common pathogens. Bacterial infections were found in 9 of 95 cases (9%), while fungal infections accounted for only 2 cases (2%). In 22 patients, no pathogen was identified. A total of 85 of 141 patients (60%) were initiated on ECMO prior to transfer from external hospitals. Detailed data are presented in Table 2. Information about non-neurological complications is summarized in Supplementary Table 2.

**Table 2:** ECMO-related patient data.

Item	Calculation
Setting (N, %)	
veno-venous	119/141 (84)
veno-arterial	22/141 (16)
Indication (N, %)	
Pneumonia	95/141 (67)
Viral	62/95 (65)
COVID	46/62 (74)
Influenza A	14/62 (23)
Influenza B	2/62 (3)
Bacterial	9/95 (9)
*Legionella pneumophila*	5/9 (56)
*Mycobacterium tuberculosis*	1/9 (11)
*Streptococcus pneumoniae*	1/9 (11)
Others	2/9 (22)
Mycotic	2/95 (2)
*Pneumocystis jirovecii*	2/2 (100)
Unknown	22/95 (23)
Aspiration	7/141 (5)
Exacerbated Chronic Obstructive Lung Disease	4/141 (3)
Trauma	5/141 (4)
Cardiogenic shock	4/141 (3)
Extracorporeal life support	16/141 (11)
Other	10/141 (7)
Start of ECMO therapy (N, %)	
ex domo	85/141 (60)
in domo	56/141 (40)
Cannulation site (N, %)	
veno-venous	
Bifemoral	64/119 (54)
Femero-jugular	33/119 (28)
Unknown	22/119 (18)
veno-arterial	
Bifemoral	15/22 (68)
Femoral (unilateral)	4/22 (18)
Unknown	3/22 (3)

ECMO, extracorporeal membrane oxygenation; N, number.

### 3.3 Intracranial complications and early neurological outcomes

Intracranial complications during ECMO therapy were frequent and included intracranial hemorrhage with intracerebral hemorrhage (51/141, 36%), subarachnoid hemorrhage (30/141, 22%), or subdural hematoma (5/141, 4%), diffuse hypoxic cerebral edema (35/141, 25%), and cerebral ischemic stroke (20/141, 14%). As expected, all patients with cerebral edema identified in this screening exhibited diffuse, bihemispheric hypoxic edema. Intracranial complications were typically discovered within 5 ± 8 days after ECMO initiation. Over half of the cases (75/141, 53%) exhibited multifocal rather than focal pathology. Imaging-based deterioration (defined as an increase in hemorrhage, infarct, or edema volume, or as the occurrence of additional intracranial complications) was observed in 59% (83/141) of patients. Over time, a midline shift developed in 28 cases (20%), and pupillary abnormalities were noted in 55 cases (39%). Neurosurgical interventions were performed in a minority of cases (29/141, 21%). Most patients were transferred to other facilities for weaning or rehabilitation with a mRS-score of 5, indicating severe disability at the time of discharge. Detailed data are presented in Table 3.

**Table 3:** Intracranial complication, neurosurgical management neurological outcomes, and non-neurological complications.

Item	Calculation
Main neurosurgical procedure (N, %)	
- External ventricular drain	9/29 (31)
- Craniectomy	20/29 (69)
Early neurological complications (N, %)	
- Intracerebral hemorrhage	51/141 (36)
- Subarachnoid hemorrhage	30/141 (21)
- Subdural hematoma	5/141 (4)
- Cerebral infarction	20/141 (14)
- Cerebral edema	35/141 (25)
Discovery of early neurological complications after ECMO therapy initiation (days, mean ± SD)	5 ± 8
Intracranial localization (N, %)	
- Focal	66/141 (47)
- Diffuse	75/141 (53)
Bleeding progression or additional neurological complication (N, %)	83/141 (59)
Intracranial midline shift at any time (N, %)	28/141 (20)
Pupillary abnormalities at any time (N, %)	55/141 (39)
Neurological outcome at discharge (N, %)	
- mRS 1	1/33 (3)
- mRS 2	0/33 (0)
- mRS 3	3/33 (9)
- mRS 4	13/33 (39)
- mRS 5	16/33 (48)
Non-neurological complications (N, %)	
- Mesenteric Ischemia	5/141 (4)
- Pulmonary embolism	28/141 (20)
- Endobronchial hemorrhage	9/141 (6)
- Retroperitoneal hematoma	6/141 (4)
- Gastrointestinal bleeding	17/141 (12)
- Myocardial infarction	20/141 (14)
- DIC	13/141 (9)
- HIT	2/141 (1)
- Thrombocytopenia	57/141 (40)

ECMO, extracorporeal membrane oxygenation; N, number; SD, standard deviation; mRS = Modified Rankin Scale Score; DIC, Disseminated intravascular coagulation; HIT, Heparin-induced thrombocytopenia.

### 3.4 Predictors of in-hospital mortality during ECMO therapy

While in-hospital mortality was 59% in patients without intracranial bleeding complications, it increased to 77% in those with such complications (OR = 2.275; 95% CI = 1.494 – 3.465; p < 0.001). Univariate analysis identified secondary deterioration (OR = 2.835; 95% CI = 1.271 – 6.321; p = 0.009), midline shift (OR = 10.667; 95% CI = 1.391 – 81.824; p = 0.005), and pupillary abnormalities (OR = 14.936; 95% CI = 3.404 – 65.539; p < 0.001) as significantly associated with in-hospital mortality. The results of the univariate analyses are summarized in Table 4. However, in the multivariate analysis, only pupillary abnormalities remained a significant predictor (aOR = 11.499; 95% CI: 2.010 – 65.786, p = 0.006). In contrast, bleeding progression or additional neurological complications (aOR = 1.739; 95% CI: 0.726-4.164; p = 0.214) and intracranial midline shift at any time (aOR = 1.289; 95% CI: 0.106-15.618; p = 0.842) were not significantly associated with in-hospital mortality (Supplementary Table 1).

**Table 4:** Predictors of in-hospital mortality. Univariate.

Item	Survivor (N = 33)	Non-survivor (N = 108)	p-value	OR	95% CI
Female sex (N, %)	18 (55)	53 (49)	0.691^b^	0.803	0.367-1.756
Age (years, mean ± SD)	45.2 ± 16.8	48.9 ± 14.1	0.214^a^	N/A	N/A
Height (cm, mean ± SD)	170 ± 11	172 ± 11	0.318^a^	N/A	N/A
Weight (kg, mean ± SD)	83 ± 25	94 ± 31	0.066^a^	N/A	N/A
BMI (kg/m^2^, mean ± SD)	28 ± 7	32 ± 9	0.052^a^	N/A	N/A
Active smoker (N, %)	4 (12)	18 (17)	0.784^b^	1.450	0.454-4.632
CCI (mean ± SD)	1.5 ± 1.6	2.1 ± 1.8	0.130^a^	N/A	N/A
SAPS_peak_ (mean ± SD)	46.4 ± 13.0	51.2 ± 16.6	0.135^a^	N/A	N/A
Veno-venous ECMO therapy (N, %)	30 (91)	89 (82)	0.286^b^	0.468	0.129-1.695
Indication (N, %)					
- Pneumonia	23 (70)	72 (67)	0.745^b^	0.870	0.374-2.021
- Aspiration	3 (9)	4 (4)	0.354^c^	0.385	0.082-1.814
- Exacerbated COPD	2 (6)	2 (2)	0.233^c^	0.292	0.040-2.162
- Trauma	1 (3)	4 (4)	0.999^c^	1.231	0.133-11.410
- Cardiogenic shock	2 (6)	2 (2)	0.233^c^	0.292	0.040-2.162
- ECLS	1 (3)	15 (14)	0.085^b^	5.161	0.655-40.646
Start of ECMO therapy ex domo (N, %)	22 (67)	63 (58)	0.424^b^	0.700	0.309-1.587
Neurosurgical procedure (N, %)	5 (15)	24 (22)	0.467^b^	1.600	0.558-4.591
Early neurological complications (N, %)					
- Intracerebral hemorrhage	9 (27)	42 (39)	0.224^b^	1.697	0.719-4.003
- Subarachnoid hemorrhage	9 (27)	21 (19)	0.336^b^	0.644	0.261-1.587
- Subdural hematoma	1 (3)	4 (4)	0.999^c^	1.231	0.133-11.410
- Cerebral infarction	4 (12)	16 (15)	0.999^c^	1.261	0.390-4.073
- Cerebral edema	10 (30)	25 (23)	0.405^b^	0.693	0.291-1648
Discovery of early neurological complications after ECMO therapy initiation (days, mean ± SD)	6 ± 9	4 ± 8	0.293^a^	N/A	N/A
Diffuse Intracranial localization (N, %)	18 (55)	57 (53)	0.859^b^	0.931	0.426-2.037
Secondary radiographic deterioration or additional intracranial complication (N, %)	13 (39)	70 (65)	**0.009^b^**	2.835	1.271-6.321
Intracranial midline shift at any time (N, %)	1 (3)	27 (25)	**0.005^c^**	10.667	1.391-81.824
Pupillary abnormalities at any time (N, %)	2 (6)	53 (49)	**<0.001^b^**	14.936	3.404-65.539
Non-neurological complications (N, %)					
- Mesenteric Ischemia	1 (3)	4 (4)	0.999^c^	1.231	0.133-11.410
- Pulmonary embolism	4 (12)	24 (22)	0.318^c^	2.071	0.663-6.474
- Endobronchial hemorrhage	1 (3)	8 (7)	0.685^c^	2.560	0.308-21.256
- Retroperitoneal hematoma	0	6 (6)	0.336^c^	N/A	N/A
- Gastrointestinal bleeding	3 (9)	14 (13)	0.762^c^	1.489	0.401-5.536
- Myocardial infarction	5 (15)	15 (14)	0.784^c^	0.903	0.602-2.705
- DIC	2 (6)	11 (10)	0.733^c^	1.758	0.369-8.364
- HIT	0	2 (2)	0.999^c^	N/A	N/A
- Thrombocytopenia	12 (36)	45 (42)	0.587^b^	1.250	0.558-2.798

N, number; SD, standard deviation; BMI, Body-Mass-Index; CCI, Charlson Comorbidity Index; SAPS_peak_, Peak of Simplified Acute Physiology Score; ECMO, extracorporeal membrane oxygenation; ECLS, extracorporeal life support; COPD, Chronic Obstructive Lung Disease; DIC, disseminated intravascular coagulation; HIT, heparin-induced thrombocytopenia; OR, odds ratio; 95% CI, 95% confidence interval; ^a^ Student's t-test; ^b^ Chi-Square test; ^c^ Fisher exact test.

### 3.5 Subgroup analysis

**Fig. 1:**
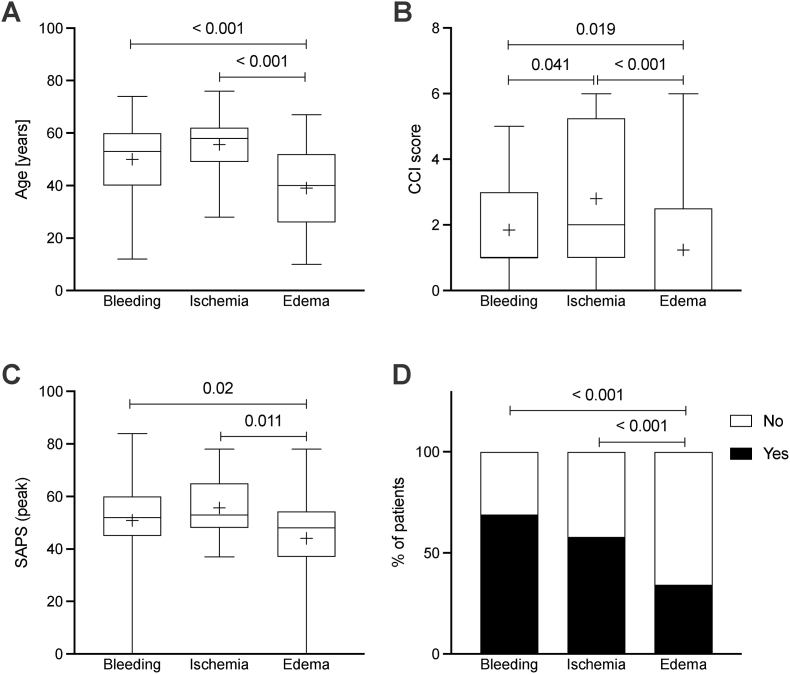
**Subgroup analysis**. The subgroups bleeding, ischemia, and edema were analyzed. (A) Age in years, (B) CCI, Charlson Comorbidity Index, and (C) SAPSpeak, Peak of Simplified Acute Physiology Score, of the respective patient groups. Data are presented as box plots. Kruskal-Wallis test was applied. The plus sign indicates the mean. Only significant p-values are shown. (D) Proportion of patients with an increase in the size of their intracranial pathology and/or the occurrence of a secondary intracranial pathology within each patient group. Percentages are displayed. Chi-square test was applied.

The cohort was stratified into three subgroups: bleeding, ischemic stroke, and diffuse hypoxic cerebral edema. A Kruskal-Wallis test revealed significant differences in age, CCI, SAPS scores, and incidence of secondary intracranial complications across groups (Fig. 1). Patients with edema were significantly younger (39 ± 15 years) than those with bleeding (50 ± 14 years; p < 0.001) or ischemic stroke (56 ± 12 years; p < 0.001) (Fig. 1A). They also had lower multimorbidity, reflected in a lower CCI score (1.34 ± 1.89), compared to bleeding patients (1.93 ± 1.56; p = 0.019) and significantly lower than ischemic stroke patients (3.00 ± 2.11; p = < 0.001) (Fig. 1B). Patients with edema had lower SAPS scores (44.0 ± 18.3) compared to those with bleeding (50.9 ± 15.4; p = 0.02) and significantly lower than ischemic stroke patients (55.6 ± 10.9; p = 0.011) (Fig. 1C). Secondary intracranial bleeding complications were less frequent in patients with diffuse hypoxic brain edema (34.3%) than in those with bleeding (69%; p < 0.001) or ischemic stroke (58%; p < 0.001) (Fig. 1D).

**Fig. 2:**
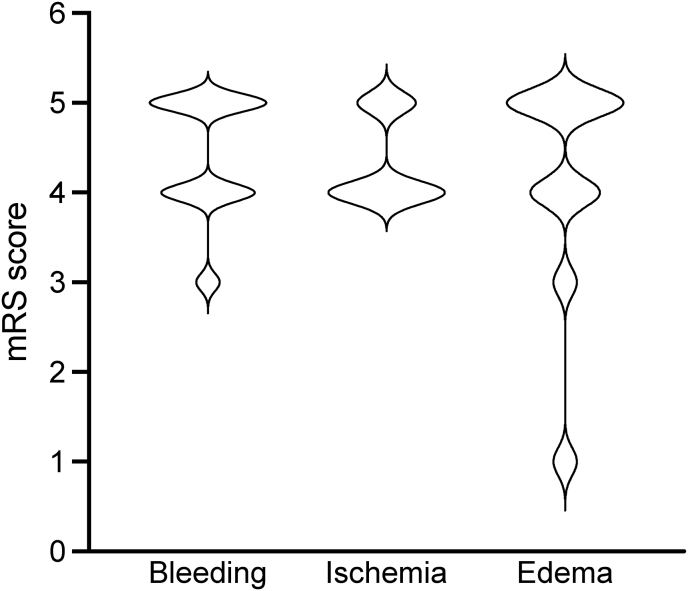
**Functional neurological outcome at discharge**. The subgroups bleeding, ischemia, and edema are shown. Data are presented as a violin plot. Only patients who survived the treatment were included, excluding those classified as mRS 6.

Despite these differences, in-hospital mortality remained high across all groups: edema (71%), bleeding (78%), and ischemic stroke (80%), with no significant intergroup differences (p = 0.746). Among survivors, neurological outcomes at discharge, measured by the mRS score, were similarly poor across all groups: cerebral edema (4.10 ± 1.29), bleeding (4.37 ± 0.68), and ischemic stroke (4.50 ± 0.58; p = 0.999) (Fig. 2).

## 4 Discussion

ECMO is a life-saving intervention for patients with severe cardiac or respiratory failure, serving as a bridge to recovery, transplant, destination therapy, or further decision-making (Haas et al., 2022). However, despite advanced hemodynamic and organ monitoring, neurological assessment during ECMO remains a challenge. This is largely due to sedation, the complexity of underlying conditions, and frequent hemodynamic instability (Fletcher-Sandersjöö et al., 2018; Cho et al., 2024). At the same time, ECMO patients face a high risk of intracranial complications, which are known to significantly impact prognosis (Nasr and Rabinstein, 2015; Nunez et al., 2022). This underscores the importance of evaluating intracranial events during ECMO therapy - not only to understand their incidence but also to derive potential therapeutic implications.

Previous studies have reported a high incidence of intracranial complications in ECMO patients but often provide limited data on mortality and neurological outcomes. While the literature suggests an incidence of approximately 16% (Shoskes et al., 2020), detailed assessments of in-hospital mortality and functional outcomes remain scarce. Furthermore, existing studies often combine different intracranial complications into one analysis, use small sample sizes, and include heterogeneous patient populations - factors that limit the generalizability and reliability of their findings.

Our study adds to the existing literature by providing data from a large, well-characterized patient cohort treated according to a standardized protocol at a tertiary care center across pre-COVID, COVID, and post-COVID periods. Compared to the largest systematic review by Shoskes et al. (2020), our cohort is notable for its size, consistent management, and comprehensive evaluation of intracranial complications.

The overall in-hospital mortality rate of 62% aligns with expectations and is comparable to findings from previous studies (Herrmann et al., 2022; Hilder et al., 2017; Shoskes et al., 2020; Enger et al., 2017). Patients with intracranial complications exhibited a higher mortality rate of 77%, consistent with reports from other authors (Prinz et al., 2021).

We observed an incidence of intracranial complications of 18%, which aligns well with previously published data (Nasr and Rabinstein, 2015). The distribution of specific pathologies - bleeding, stroke, and diffuse hypoxic brain edema - was also consistent with prior reports (Fletcher-Sandersjöö et al., 2018; Nasr and Rabinstein, 2015), supporting the external validity of our findings. Slightly differing rates in other studies may reflect differences in ECMO modality (veno-venous vs. veno-arterial), patient populations (e.g., COVID-19-specific cohorts), or the lack of standardized definitions for intracranial bleeding (intracranial vs. intracerebral) and inconsistent imaging protocols (Nunez et al., 2022; Lüsebrink et al., 2022; Lannon et al., 2023; Lockie et al., 2017).

Our univariate analysis revealed that the presence of secondary deterioration on cranial CT, midline shift, and pupillary abnormalities significantly increased the likelihood of in-hospital mortality. Notably, in the multivariate analysis, pupillary abnormalities emerged as an independent predictor of death. These findings are not only statistically significant but also clinically plausible. ECMO patients are particularly vulnerable to septic complications and are routinely anticoagulated, both of which can heighten the risk of intracranial pathology. It is well established that neuroinflammation and coagulation disorders contribute to the pathophysiology of intracranial hemorrhage (Qin et al., 2022), and these mechanisms may underlie the deterioration observed in our cohort. Midline shift and pupillary abnormalities, in particular, are key indicators of elevated intracranial pressure and the displacement of cerebral structures. While a midline shift can often be temporarily compensated by the brain, the appearance of pupillary abnormalities signals a critical decline - typically reflecting compromised brainstem perfusion. In such cases, urgent neurosurgical intervention is essential to prevent irreversible damage or death (Griepp et al., 2024). Importantly, pupillary abnormalities should not be interpreted as an early prognostic or screening marker but rather as a manifestation of already advanced intracranial injury and impending brainstem compromise. Accordingly, our data demonstrate an association with mortality rather than usefulness for early detection. We therefore cannot draw conclusions regarding their value as a screening parameter, and clinical experience suggests that their predictive utility at earlier stages of neurological deterioration is limited. This issue should be explored further in a dedicated study.

Surprisingly, the SAPS score - which is known to negatively correlate with survival in many studies on intensive care patients - was not associated with survival probability in our cohort (Le Gall, 1993). This may be attributable to the limited sample size and, potentially, to methodological factors in the calculation of SAPS scores.

Given the heterogeneity of ECMO patients, differentiation between bleeding, ischemic stroke, and diffuse hypoxic cerebral edema is essential. Our data indicate that these entities differ not only in pathophysiology but also in patient characteristics and outcomes. However, these differences did not translate into meaningful differences in mortality or functional neurological outcome, both of which remained poor across all groups. Consequently, the present findings should not be interpreted as evidence for clinically relevant prognostic stratification but rather as an indication that distinct mechanisms may lead to a similarly unfavorable clinical course. For example, patients with edema were typically younger, healthier, and less likely to experience secondary deterioration compared to those with bleeding or stroke. Although mortality was high across all groups, our findings suggest that each pathology should be considered individually. Notably, the observed differences may reflect their distinct underlying mechanisms: ischemic stroke often results from thromboembolic events (Aitbaev et al., 2023), bleeding during ECMO is usually due to the interplay of anticoagulation, inflammation, and hemodynamic instability (Cai et al., 2023), and edema may arise from fluid overload, hypoxemia, or cerebral autoregulatory failure (Illum et al., 2021).

Intracranial complications were closely associated with poor neurological outcomes at discharge. Nearly half of the affected patients had a score of 5 on the mRS, while only 12% achieved a favorable outcome with a score of 3 or lower. This rate is lower than what is typically reported for patients undergoing ECMO therapy, suggesting a substantial impact of intracranial complications on the functional neurological outcomes of survivors (Kalra et al., 2024). It is important to note that discharge from our center marks an early point in the recovery process. Most patients were transferred to rehabilitation facilities or external weaning centers, so long-term neurological outcomes could not be assessed. Studies on functional recovery and quality of life after intracranial events are planned at our center.

Our study has several limitations, including its retrospective nature, the single-center design, and the inability to assess long-term outcomes. Additionally, neurological status at discharge may underestimate eventual recovery, as ECMO survivors can continue to improve beyond discharge. Moreover, our study does not provide information on risk factors for the occurrence of intracranial complications, as our analysis was limited to patients who had already developed intracranial complications. Future research addressing this aspect would be valuable to identify predictors of at-risk patients and to evaluate strategies for early risk assessment. An additional limitation is that follow-up cranial CT imaging was performed based on clinical indications rather than according to a predefined imaging protocol. Consequently, clinically silent intracranial complications may have remained undetected, potentially resulting in an underestimation of the true incidence of intracranial complications and introducing a detection bias. Moreover, the generalizability of our findings may be limited, as established ECMO retrieval programs are not universally available across healthcare systems. Consequently, patient selection, timing of ECMO initiation, and transfer logistics may differ substantially between institutions and regions. Nevertheless, our dataset is well-characterized and represents a homogeneously treated cohort, offering valuable insights into a critical and under-researched complication of ECMO therapy. Given that intracranial complications are often advanced by the time they are clinically detectable, we propose a reevaluation of current monitoring strategies. Early detection of rising intracranial pressure in high-risk patients may open therapeutic windows. A recent international consensus panel recommended several neuromonitoring tools, including near-infrared spectroscopy, transcranial Doppler ultrasound, automated pupillometry, electroencephalography, evoked potentials, and serum biomarkers. However, the panel discouraged the routine use of early external ventricular drainage due to the high bleeding risk associated with systemic anticoagulation during ECMO (Cho et al., 2024). In light of newer, minimally invasive intracranial pressure monitoring methods and the possibility of temporarily pausing anticoagulation in selected cases, we believe - based on interdisciplinary anesthesiology and neurosurgical expertise – that early intervention should be considered in high-risk patients. The challenge lies in identifying such patients.

Therefore, further research is urgently needed to identify predictors of intracranial hemorrhage, ischemic stroke, and cerebral edema during ECMO therapy. Given the relatively low incidence of individual neurological complications and the heterogeneity of ECMO populations, such efforts will likely require large multicenter datasets and established international platforms such as the Extracorporeal Life Support Organization (ELSO) Registry.

## 5 Conclusion

Intracranial complications during ECMO therapy are frequent, clinically diverse, and strongly associated with high in-hospital mortality and poor neurological outcomes. Pupillary abnormalities in patients with intracranial pathology emerged as the most reliable predictor of death, underscoring the need for vigilant neurological assessment. Despite differences in age, comorbidity, and clinical presentation, patients with bleeding, stroke, or cerebral edema all showed similarly poor outcomes, emphasizing the severity of these events regardless of subtype. Given the often-delayed detection of intracranial complications due to sedation and monitoring limitations, earlier and more targeted neuromonitoring in high-risk patients may offer a therapeutic window. Minimally invasive techniques and individualized anticoagulation strategies should be further explored to improve safety and outcomes. Future studies are needed to refine risk stratification and evaluate long-term recovery after ECMO-associated neurological injury.

## Ethics approval and consent to participate

This study was conducted in accordance with the Declaration of Helsinki and was approved by the local ethics committee of the University Hospital Essen (reference number: 24-120884-BO, 10.10.2024). Due to the retrospective nature of the study and the anonymization of all data, informed consent was not required. The study is registered in the German Clinical Trials Register (DRKS00035277).

## Consent for publication

Not applicable.

## Availability of data and materials

The datasets used and/or analyzed during the current study are available from the corresponding author on reasonable request.

## Authors' contributions

The study was conceptualized by JW and LR. Study design was developed by JW, LR, FH, US, and TB. Clinical data were collected by JW and FI, while imaging data were acquired and analyzed by LR, FG, MDO and JR. All imaging findings were independently reviewed and validated by CD. Data analysis and interpretation were performed by JW, LR, and BS. Statistical analysis was conducted by JW, LR, and BS. Project supervision was provided by FH, RJ, US, and TB. The original manuscript draft was written by JW and LR, with all authors contributing to the manuscript revision. JW, FI, FG, JR, RJ, MDO, BS, CD, FH, TB, US and LR approved the final version of the manuscript.

## Sources of funding

Open Access funding enabled and organized by Projekt DEAL.

## Declaration of competing interest

The authors declare that they have no known competing financial interests or personal relationships that could have appeared to influence the work reported in this paper.
