# Supplementary material for: Incidence and impact of intracranial complications in patients undergoing extracorporeal membrane oxygenation treatment
Source: Brain Spine. 2026 Jun 11;6:106123. doi: 10.1016/j.bas.2026.106123 (PMC13316211; doi:10.1016/j.bas.2026.106123)
Supplement: Multimedia component 1 [file mmc1.docx]

**Supplementary table 1. Predictors of in-hospital mortality. Multivariate.**

| **Item** | **p-value** | **aOR** | **95 % CI** |
| --- | --- | --- | --- |
| Bleeding progression or additional neurological complication | 0.214 | 1.739 | 0.726-4.164 |
| Intracranial midline shift at any time | 0.842 | 1.289 | 0.106-15.618 |
| Pupillary abnormalities at any time | 0.006 | 11.499 | 2.010-65.786 |

aOR, Adjusted Odds Ratio; 95% CI, 95% confidence interval.

**Supplementary table 2. Non-neurological complications.**

| Non-neurological complications (N, %)  - Mesenteric Ischemia  - Pulmonary embolism  - Endobronchial hemorrhage  - Retroperitoneal hematoma  - Gastrointestinal bleeding  - Myocardial infarction  - DIC  - HIT  - Thrombocytopenia | 5/141 (4)  28/141 (20)  9/141 (6)  6/141 (4)  17/141 (12)  20/141 (14)  13/141 (9)  2/141 (1)  57/141 (40) |
| --- | --- |

N, number; DIC, Disseminated intravascular coagulation; HIT, Heparin-induced thrombocytopenia.
